# Experimental Findings and Validation on Torsional Behaviour of Fibre-Reinforced Concrete Beams: A Review

**DOI:** 10.3390/polym14061171

**Published:** 2022-03-15

**Authors:** Paul Oluwaseun Awoyera, John Uduak Effiong, Oladimeji Benedict Olalusi, Krishna Prakash Arunachalam, Afonso R. G. de Azevedo, Flavia R. B. Martinelli, Sergio Neves Monteiro

**Affiliations:** 1 Department of Civil Engineering, Covenant University, Ota 112233, Nigeria; paul.awoyera@covenantuniversity.edu.ng (P.O.A.); john.effiongpgs@stu.cu.edu.ng (J.U.E.); 2 Structural Engineering & Computational Mechanics Group (SECM), Department of Civil Engineering, University of KwaZulu-Natal, Durban 4001, South Africa; olalusio@ukzn.ac.za; 3 Department of Civil Engineering, Anna University, Chennai 600025, India; krishnaprakash3191@gmail.com; 4 LECIV-Civil Engineering Laboratory, UENF-State University of the Northern Rio de Janeiro, Av. Alberto Lamego, 2000, Campos dos Goytacazes 28013-602, Brazil; 5 LAMAV-Advanced Materials Laboratory, UENF-State University of the Northern Rio de Janeiro, Av. Alberto Lamego, 2000, Campos dos Goytacazes 28013-602, Brazil; flavia.vix2014@gmail.com; 6 Department of Materials Science, IME—Military Institute of Engineering, Square General Tibúrcio, 80, Rio de Janeiro 22290-270, Brazil; snevesmonteiro@gmail.com

**Keywords:** fibres, reinforced concrete, failure mechanisms, torsional behaviour, green concrete

## Abstract

Fibres have long been utilized in the construction sector to improve the mechanical qualities of structural elements such as beams, columns, and slabs. This study aims to review the torsional behaviour of various forms of fibre reinforced concrete to identify possible enhancements and the practicability of concrete structural beams. Concrete reinforced steel fibre, synthetic fibre, and hybrid fibre are examples of fibre reinforced concrete. The review found that the mixing, orientation, and volume of fibres, the size of coarse particles, the aspect ratio of fibres, and the stiffness of fibres all affect the torsional strength of fibre reinforced concrete. Nevertheless, the application of fibres to recycled self-consolidating concrete of various forms needs to be explored and studied to ascertain its feasibility to facilitate greener concrete. Thus, with the results compiled in this review paper, it was possible to delimit advances and gaps on the effect of editing reinforcement fibres in relation to the torsion of structural elements.

## 1. Introduction

Concrete is arguably the most extensively utilized building material in construction [1]. However, it faces potential problems, such as seismic damage and cracking due to shrinkage and expansion [2]. This deficiency, when incurred, enables moisture to enter the structure, resulting in reinforcing bar corrosion and expansion, as well as a loss of structural integrity [3].

Although torsional stresses occur in many reinforced concrete buildings, design engineers mostly ignored torsion prior to the 1960s. It was anticipated that torsional impacts were small and that the substantial safety factors utilized in flexural design would take care of them. Much torsional strain and failure incidents have resulted from this notion [4].

Fibre reinforced concrete is becoming popular as a viable solution to increase concrete performance. The fibre is now used for tunnelling, bridge decks, flooring, docks loading, thin unbounded overlays, concrete pads, and concrete sheets. These fibre reinforced concrete solutions are gaining popularity and demonstrating great performance [5]. Fibre reinforced composite concrete is a cement, sand, coarse aggregate composite material containing the right proportion of fibres that are evenly dispersed, discontinuous, and distinct. In other words, it is a type of concrete that contains fibres [6,7]. Using a fibrous material improves the material’s structural integrity [3,5].

Fibres in cementitious materials have become popular, especially in constructions that require performance improvement, resulting in the application of reinforced concrete in more extreme situations [1]. The wide variety of technical standards around the world, related to the validation of reinforced structures, have led to studies related to the use and application of fibres in concrete [2]. In addition to synthetic fibres, which are most commonly used to reinforce structural elements, the use and application of natural fibres has been diversifying, especially in countries that have an abundance of these materials, such as Brazil and India [6,7]. On the other hand, the use and development of a mixture of natural and artificial fibres may be promising, especially in evaluating torsion parameters in reinforced elements.

**Figure 1:**
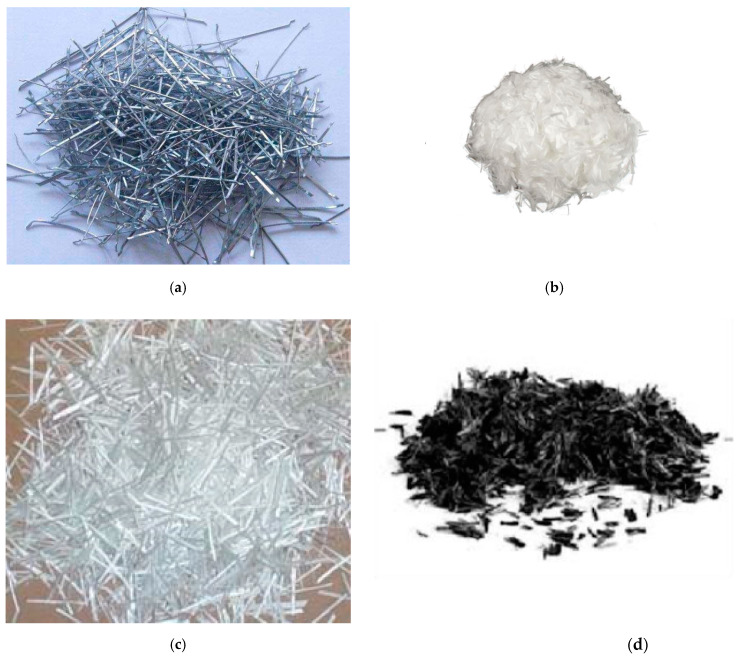
Different fibres: (**a**) Steel fibres; (**b**) Polypropylene fibres; (**c**) Glass fibres; (**d**) Carbon fibres.

Fibre reinforced concrete comes in various forms with different properties, each with its own advantages. Long wires, continuous meshes, woven textiles, or rebars, are not considered discrete fibres [8]. A fibre is a small fragment of reinforcing material with tensile strength properties. Fibres might be flat or round. The fibre is frequently characterized by the parameter of “Aspect ratio” [9]. The fibre’s aspect ratio is the proportion of its length to its diameter. The aspect ratio varies between 30 and 150. Steel fibres, glass fibres, polypropylene fibres, carbon fibres and organic fibres are examples of fibres; examples are presented in Figure 1.

With varied concretes, fibre materials, geometries, distribution, orientation, and densities, the character of fibre reinforced concrete changes within these distinct fibres. Fibres as a composite enhancer in terms of structural resilience are applied very commonly in shotcrete, although they may also be utilized in conventional concrete of a reinforced state [10]. Fibres, such as short hair-shaped glass fibres, tend to be useful for the initial few hours after the fresh concrete mix is poured (while the concrete stiffens, they reduce cracks). However, they do not raise the structural capacity of the concrete [11].

The physical parameters of the fibres are extremely important in their immersion to reinforce cementitious matrices. The length of the fibres, for example, is an important element related to anchoring, as well as the surface behaviour and interfacial interaction between the reinforcement (fibre) and the matrix (concrete), which impacts on the technological properties of the parts, such as in relation to torsion and general mechanical strength [1,8]. Torsion in concrete elements is little explored in the literature in general and has been considered secondary in several structural design processes, which justifies the research studies and literature on the subject.

**Figure 2:**
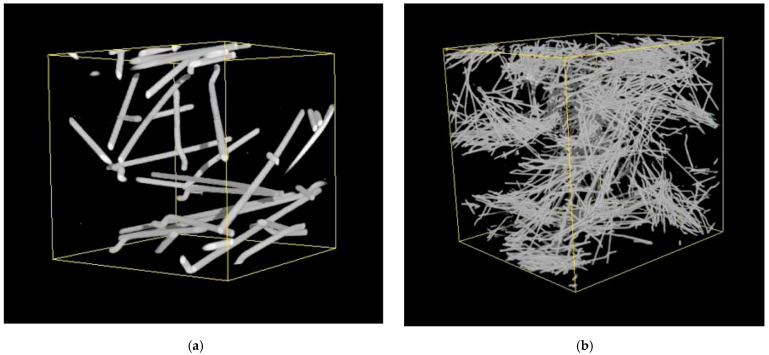
Fibre volumetric distribution in concrete specimens (**a**) containing steel fibres with length of 60 mm; (**b**) containing steel fibres with length of 13 mm [14].

Fibre in concrete improves the concrete’s tensile strength. It reduces the intrinsic porosity of gel by reducing air and water gaps. It boosts the concrete’s durability. Most resins do not have the same creep resistance as fibres such as graphite and glass [12]. As a result, the volume fraction and orientation of the fibres has a major impact on the creep behaviour of tendons/reinforcement bars though the distribution, orientation, and segregation of fibres all of which are influenced by the mixing, casting, and vibration methods utilised [13]. Some important techniques were applied to measure the amount of steel fibres in concrete specimens using an X-CT, showing the strong influence of the volume fraction of steel fibres on the different technical properties of steel reinforced concretes. Figure 2 shows comparative images of the distribution of steel fibres in two different specimens with 40 mm × 40 mm × 40 mm dimensions [14].

Concrete reinforced with steel is a composition of materials in which the rebars serve as the strengthening fibre, and the concrete serves as the matrix. Therefore, it is also critical that these two materials behave similarly under thermal loads to reduce concrete and reinforce non-linear deflections. It has long been understood that tiny, closely spaced, evenly dispersed fibres act as crack arrestors when added to concrete, as they increase both crack strength and ultimate strength [15]. In other words, they act as fracture arresters and significantly improve the concrete’s static and dynamic qualities.

Researchers have been studying the behaviour of reinforced concrete structures under torsional moments for several decades. After the pioneering Graf and Morsch [16] experiments, several studies on the structural performance of concrete structures subjected to torsional moments or in combination with other activities have been described in the literature. Because of the rising utilization of high-performance concretes in research and applications in previous years, investigations on regular reinforced concretes with compressive strengths beyond 100 N/mm^2^ have been reported [17]. Reinforced concrete elements such as beams reinforced with materials of the future which have been subjected to torsional moments have also in recent times been studied [18,19]. These studies reveal that following the first cracking, the shear loads caused by the produced torsion cannot be withstood unless a suitable mechanism which allows stress to be transmitted across a damaged region is introduced. One such process may be explained using the space truss analogy, which was first proposed by Rausch [20,21] and then developed by Lampert [22] and Lampert and Collins [23]. These models frequently include diagonal compressive struts and tie components (such as links, transverse and longitudinal reinforcement bars) that are utilized to restore balance and transmit loads inside the cracked sections [24]. Moreover, multivariable regression strength models and other analytical/numerical models for SFRC under torsion have been considered in recent investigations. Deifalla et al. [25] proposed a twofold model based on the optimization of constants obtained from formulations which are already available by applying multi-linear regression. In addition, another model was developed based on modifying the American Concrete Institute (ACI) design code for reinforced concrete (RC) members, which took into consideration the effect of steel fibres on the torsional capacity of SFRC beams. The study showed that the model exhibited strong predictions. A model for torsional behaviour of SFRC considering the percentages of longitudinal steel reinforcement and the proportions of recycled aggregates was proposed by Chkheiwer et al. [26]. Their model showed that beams with maximum steel enhancement and standard aggregate exhibited maximum cracking power and ultimate torsional strength.

As first discovered by Leonhardt and Monning [27] and later indicated by numerous structural codes, structural concrete must have a minimum area of steel for longitudinal and transverse rebars to mitigate fragile failure modes at the start of initial cracking [28]. Given that the role provided by rebars in torsion-resistant structural elements is similar to that of shear links in shear-resistant beam structures, the research findings presented by Choi et al. [29], Susetyo et al. [30], Minelli and Plizzari [31], and Facconi and Minelli [32] show promising findings which support the idea of partially or completely creating an alternative for the minimum transverse reinforcement. Steel fibres that are randomly oriented and equally scattered improve the post-cracking performance of concrete elements.

In recent research conducted globally, fibres have been successfully employed in various categories to improve the performance of structural elements such as beams in various forms [33,34,35,36,37,38,39], tunnel linings [40,41,42], slabs [43,44] and other flexural- and shear-critical members. 

Civil infrastructures provide users with excellent functions; yet, they are subject to degradation, hence all the different forms of stresses on the structure have to be taken into consideration during analysis and design for improved durability [45]. Structure engineers generally design the RC beam load without consideration for torsional moments and hence, in structural analyses, the torsion impact is frequently disregarded. Torsional effects rarely occur alone [46]. It is a core stress resultant which is required to be taken into account when designing structural beams, bridge girders, curved components, and so on [37]. These torsional moments can increase strains on the member and alter the entire structure’s behaviour. Eccentric loads are normally avoided in beam construction, although they are quite common in bridges and induce a combination of bending, axial, torsion and shearing stresses inside the structural elements. The tandem impact of torsional moments, flexure, and shear on a beam experiencing unusual stress has scarcely been referenced experimentally in research; thus, achievements are not as substantial as they appear in the areas of bending, shear, and torsion separately. Similarly, the strengthening choices for flexural shear and torsion are accessible separately; however, combining the effects of torsion, flexural, and shear strengthening choices is a crucial area of study [45]. 

This study aims to review the torsional behaviour of various forms of fibre reinforced concrete to identify possible enhancements and the practicability of concrete structures. The innovation of this research is the limited comprehensive and compiled literature on the torsional behaviour of fibre reinforced concrete elements, especially in relation to the wide variability of technical standards around the world. Thus, it is expected to contribute to the discussion on the subject and the area of coverage, and aims to improve practices and various applications.

## 2. Types of Fibre Reinforced Concrete

Reinforced Concrete incorporating fibres exist in various forms such as:

- Reinforced Concrete incorporating steel fibres
- Reinforced Concrete incorporating synthetic fibres
- Reinforced Concrete incorporating hybrid fibres

### 2.1. Reinforced Concrete Incorporating Steel Fibres

A variety of steel fibre types are available and employed in concrete production. The most conventionally utilized form of steel fibre is round steel fibre. It is produced by breaking round wire into small strands. The diameter is usually from 0.25 mm to 0.75 mm. Steel fibres with a rectangular cross-section also exist and are manufactured by silting 0.25 mm thick sheets. Mild steel drawn wire is used to make the fibre. In India, wire diameters ranging from 0.3 to 0.5 mm have been utilized in accordance with IS:280-1976. Hat sheet fibres with a typical cross-section between 0.15 and 0.41 mm in thickness and between 0.25 mm and 0.90 mm in breadth are generated by silting Hat sheets with a typical cross section between 0.15 and 0.41 mm in thickness and between 0.25 and 0.90 mm in breadth. Deformed fibre can also be found in the shape of a bundle, loosely connected with water-soluble glue. Individual fibres tend cause a balling effect in the matrix, making uniform dispersion problematic. To avoid this, fibre stacks that dissociate during the mixing process might be employed.

### 2.2. Reinforced Concrete Incorporating Synthetic Fibres

#### 2.2.1. Polypropylene Fibre Reinforced (PFR) Cement Mortar and Concrete

One of the most affordable and widely used polymers of low modulus is polypropylene. The cementitious structure would disintegrate first under intense chemical assault since polypropylene fibres are resilient to many chemicals. It is characterized by a melting point of approximately 165 degrees centigrade which is considered high. This allows for a working temperature of 100 degrees Celsius to be maintained for short periods without compromising fibre qualities. Since polypropylene fibres are hydrophobic, they are easy to mix because they do not require prolonged contact and they rarely have to be uniformly disturbed in the mix. Commercially available polypropylene fibres with tiny volume fractions ranging from 0.5% to 15% are utilized in concrete mix.

#### 2.2.2. Glass Fibre Reinforced Concrete

Glass fibre consists of 200–400 individual filaments that are loosely linked together to form a strand. These strands are usually cut into different sizes or joined to create a textile fabric. It is difficult to mix more than 2% (by volume) of fibres with a length of 25 mm using traditional mixing procedures for standard concrete. Glass fibre has been chiefly employed to reinforce the shotcrete matrix used in the manufacture of thin-sheet goods. E-glass is a kind of glass fibre that is widely utilized though it has insufficient resilience to alkalis contained in Portland cement, but AR-glass has a better alkali resilience.

#### 2.2.3. Carbon Fibre Reinforced Concrete

Carbon fibres are the oldest and, by far, the most profound inclusion to the range of commercially utilized fibres. Carbon fibre has an extremely high elasticity and flexural strength modulus. However, they are expensive. Even steel’s strength and stiffness attributes have been shown to be superior. Because they are more susceptible to damage than glass fibre, they are usually coated with a resign coating.

#### 2.2.4. Asbestos Fibre Reinforced Concrete

Asbestos, a naturally occurring and affordable synthetic fibre is conventionally mixed with Portland cement paste to produce asbestos cement, a commonly utilized material. Asbestos fibres are useful for sheet product pipes, tile production, and corrugated roof parts due to their thermal, mechanical, and chemical resilience. Unreinforced matrix is around two to four times the cost of asbestos cement board. The fibre, however, has moderate impact strength due to its short length (10 mm).

#### 2.2.5. Organic Fibre Reinforced Concrete

Steel and glass fibres may be less chemically inert than organic fibres such as polypropylene or natural fibre. They are also less expensive, especially if they are natural. A considerable amount of vegetable fibre might be employed to make a multiple cracking composite. An admixture can be used to overcome the issues of mixing and uniform dispersion.

### 2.3. Reinforced Concrete Incorporating Hybrid Fibres

These are a combination of more than one fibre to improve the structural integrity of concrete. This can be a mixture of glass and steel fibres or other combinations. 

## 3. Types of Fibre Reinforced Polymers

Based on previous studies, the commonly adopted fibre reinforced polymers in reinforced concrete structures include:

- Carbon fibre reinforced polymers (CFRP)
- Glass fibre reinforced polymers (GFRP)
- Aramid fibre reinforced polymers (AFRP)
- Natural fibre reinforced polymers (NFRP)

### 3.1. Carbon Fibre Reinforced Polymers (CFRP)

The exceptional properties of carbon fibre reinforced polymer (CFRP), such as its high tensile strength, low weight, superior corrosion resistance, and fatigue strength, make it a popular choice for retrofitting strategies. To raise the flexural strength, restrict the formation of cracks, and boost the serviceability of the beams, it should be anchored to the stress zone of the concrete element. It may even be used for the remediation of steel structures [47].

### 3.2. Glass Fibre Reinforced Polymers (GFRP)

Newly developed GFRP, or glass fibre reinforced polymer, is an effective material for usage in hostile environments [48]. Rebars made of glass fibre reinforced polymer (GFRP) offer excellent tensile strength, and they have lightweight, non-corrosive, anti-fatigue, and non-magnetic properties. The shear strength of the beam may be restored [49]. GFRP failures are characterized by a reduced post cracking resistance and sliding between the rebar and the concrete matrix [50]. Glass-GFRP composite beams’ post-cracking strength and ductility have been studied, and the results show that reasonably ductile failure modes may be obtained with significant increases in strength and deformation capacity, however, this is unattainable in glass beams [51]. FRP’s low Young’s modulus and high strength means that the loading capacity of FRP’s GFRP sections is limited by excessive deformation and/or local and global buckling [52].

### 3.3. Aramid Fibre Reinforced Polymers (AFRP)

The lowest specific gravity and best tensile strength-to-weight ratio of any reinforcing fibre is found in aramid fibre, a well-known synthetic organic polymer fibre. Abrasion, corrosion, impact, chemicals, and UV radiation degradation all have a detrimental effect on the material’s properties, making it difficult to machine [52].

### 3.4. Natural Fibre Reinforced Polymers (NFRP)

Unlike synthetic fibres, the qualities of natural fibres may vary widely depending on where they come from. They also tend to have lower tensile strengths than synthetic fibres. Selected natural fibres were studied by Monteiro et al. [53], who discovered an inverse connection between fibre strength and diameter. Natural fibres may be effective when extracted in tiny and homogeneous diameters from high-strength natural fibre sources such as hemp as a reinforcing material for FRP composites. However, high strength natural fibres are lighter and cheaper than glass fibres, even if the tensile strength of natural fibres is lower. This link was investigated by Dittenber and Gangarao [54], who compared the cost per length of the different types of fibre needed to transport a certain weight. Sisal, kenaf, jute, and bamboo fibres were comparable to glass fibre. In comparison with glass and carbon fibres, natural fibres are more self-sustaining; they are low cost, eco-friendly, widespread, recyclable, easy to manufacture, and they are carbon positive since they take in more CO_2_ than they generate [55]. However, few or no research studies have been carried out in recent times to identify their torsional capacity. 

## 4. Methods Adopted by Previous Studies

The methods for the preparation of the specimens used in previous studies are summarized in Table 1, Table 2, Table 3, Table 4, Table 5 and Table 6.

## 5. Results and Discussion

### 5.1. Steel Fibre Reinforced Concrete Findings

Numerous experimental tests have been performed on fibre reinforced members subjected to torsion. The report from Amin and Bentz [37] indicated that beam samples strengthened with 8 mm and 10 mm diameter stirrups based on the torque versus angle of twist tracks, resulted in a torsional strength increase of 34 and 40 percent respectively. This transformed the failure mode from almost fragile to ductile, especially for beams strengthened by 8 mm shear ligatures of two-legged diameters.

In specimens reinforced with fibres, crack widths were much smaller and cracks formed at closer spacings. As a result of the fibres’ ability to regulate cover spalling, the stirrups were better at transferring stress across the fissures because the stirrups were able to better contain the core of the specimen by retaining the interface to the surrounding concrete matrix. This is due to improved cohesion between the steel rebars and the fibre matrix. [37].

Facconi et al. [36] experimented on six beams of equal dimensions (2700 mm in length 300 mm wide and 300 mm deep) loaded in pure torsion with a 20 mm cover according to Eurocode 2 specifications. The results from the experiment indicated that steel fibres in the mix yield greater torsional strength. Torsional strength increased by 41.85–69% [36]. 

Patil et al. [34] carried out an experimental program that entailed casting four reinforced concrete beams with dimensions of 150 mm × 150 mm and a length of 2 m. One beam was cast fibreless in order to compare it to the remaining three beams: one was cast with 0.5 percent fibre by weight, one with 1.0 percent fibre by weight, and the remaining one was cast with 1.5 percent fibre by weight. Shear stirrup spacing and longitudinal reinforcement were kept constant. The results indicate that when the proportion of steel fibre increases, the cracking torsional strength and ultimate torsional strength also increase [34].

The utilization of fibre in reinforced concrete has been proven to be quite advantageous in increasing the torsional capacity of reinforced concrete beams exposed to the torque effect. As the proportion of steel fibre increases, so does the torsional strength at first crack and the ultimate torsional strength. The torsional strength at first crack of fibre proportion 1.5% improved up to 54.85 %, representing a considerable improvement in concrete strength over a typical reinforced concrete beam. The ultimate torsional strength of FR 1.5 improved to 72.10%, a substantial improvement in concrete strength as compared to an ordinary RC beam. The brittleness of fibre reinforced concrete was reduced, and its ductility was increased. In comparison to a normal RC beam, fibre strengthening also increased beam stiffness by reducing the angle of twist of the stronger reinforced concrete beam. In other words, by increasing the load, the initial fracture pattern was detected in the fibre reinforced strengthened beams [34].

In a study conducted by Lau et al. [39], eight alkali-activated concrete beams were investigated experimentally until failure under pure torsional load. The following variables were used to make the alkali-activated concrete beams: (i) only fibres, (ii) only conventional steel reinforcement, or (iii) a combination of fibres and conventional steel rebars. The cracking torque of fibre-only beams was around 20% greater than that of conventionally reinforced beams. Compared to conventional, alkali-activated concrete (AAC) beams, “fibres-only” beams exhibited reduced post-fracture ductileness and unpredictable post fracture behaviour. On the contrary, hybrid reinforcements in these beams were shown to have more ductile post-crack behaviour in all the specimens evaluated.

**Figure 3:**
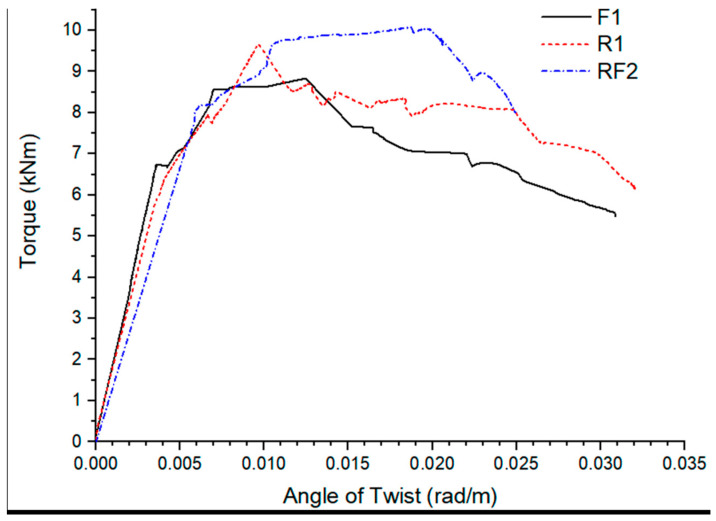
Torque vs. angle of rotation comparison of solely fibre reinforcement, conventional reinforcement, and hybrid reinforcement comprising of fibres and typical reinforcement [39].

**Figure 4:**
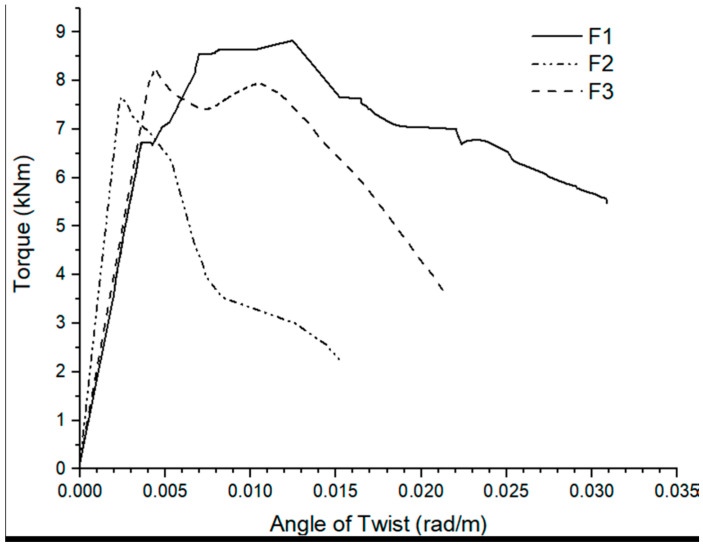
Torque vs. angle of twist for beam samples formed only with fibre reinforcement [39].

In comparison to conventionally structural elements, hybrid reinforcement improved cracking and ultimate torque by 20% and 25%, respectively, indicating that it can be suited for utilization in the construction field to increase structural capacity. The torsional moment and the twisting angle relationship is represented in Figure 3 and Figure 4.

The torsional behaviour of lightweight concrete (LWC) and normal weight concrete (NWC) was investigated in the work conducted by George and Sofi [56] which involved the manufacture of lightweight concrete, using coconut shell aggregate instead of fragmented granite. For torsional fortification, crimped steel fibres were utilized. Because torsional strength is predominantly determined by the weakest component, a torsion-loaded component which underwent torsional cracking before flexural failure was used [56]. This research demonstrated that optimal steel fibres may provide concrete with homogenous tensile characteristics, which in turn boosts its torsional strength. In George and Sofi’s study, fibre was added to NWC and LWC by 0.5%, 0.785% and 1% volume. The fundamental mechanical properties such as compressive strength, split tensile strength, and bending strength were examined for 7 days and 28 days with cubes, cylinders, and prisms. Torsional enhancement investigations on 1100 mm × 150 mm × 100 mm beams were performed. The NWC and LWC results were compared with the appropriate control mixes. There were also comparisons between the torsional characteristics of NWC and LWC. Torque-angle twist responses were investigated for all mixtures. The study indicated that LWC exhibited a greater torque value and torque angle than NWC [56].

Table 7 sets out the cracking and maximum torque of all mixtures. For all the beams the angle of twist was also measured. The graph was drawn for each mix torque vs. twist angle. Both control mixes have enhanced torsional strength by the addition of fibres.

However, as the fibre concentration ran from 0.75 to 1 percent, the torsional strength decreased marginally. In both combinations, 0.75 percent fibre demonstrated the maximum twisting resilience. Thus, 0.75% of steel fibre can be taken as the optimal fibre dose by volume. The torsional moment at which the initial crack occurred is at first cracking. The rate of torque increase in relation to the torsional angle was smaller following a cracking torque. This is the beam’s delivery step. Without significant load increases, it suffers from twisting. In relation to the control mixes of both concrete mixes, each blend with fibre has a significantly good torsional capacity. Table 7 also provides a comparison of different experimental studies for steel fibre reinforced concrete.

### 5.2. Synthetic Fibre Reinforced (SYF) Concrete

#### Polypropylene Fibre Reinforced (PFR) Cement Mortar and Concrete

Depending on the length and the purpose of polypropylene fibres, they may be separated into microfibres and macrofibres. In the study carried out by Blazy and Blazy [6], an overview of various polypropylene fibres produced commercially was presented. Furthermore, the impact of polypropylene fibres on the structural characteristics of the concrete was considered, including its elasticity modulus, workability, bending, compressive and torsional stiffness, resilience, impact, spalling, and other durability properties [6]. However, the torsional behaviour of the specimens studied were not considered. Therefore, further research into this form of fibre reinforced concrete is needed to evaluate the torsional capacity in relation to normal reinforced concrete.

Prakash et al. [5] also investigated the effect of polypropylene fibre additive on the mechanical characteristics of concrete which was tested in the form of a greener concrete made of fly-ash, an industry by-product, a cementitious material serving as a replacement material for the binder, and a coconut shell, an agro-waste. The first with the coconut shell as an alternative for coarse aggregates, the other with the mixture of conventional aggregates and coconut shell as a partial replacement for coarse aggregates were produced. A total of two alternative mixes were produced, at 10 per cent by weight in the concrete mix, the cement component was replaced by the fly ash class F. The polypropylene fibre volume fractions employed in this investigation were 0.25%, 0.5%, 0.75% and 1.0%. Adding polypropylene fibres decreases the density and slump of the coconut shell concrete marginally causing the compressive strength and elasticity modulus of the coconut shell concrete to similarly rise up to 0.5% of the fibre volume fraction as the fibre fraction grows in volume. Fibre reinforcement has also been added to the divided tensile strength and bending force of coconut shell concrete. A minor reduction in compressive strength is the inclusion of 0.75 and 1.0 per cent volume polypropylene fibres. The results of this research suggest that polypropylene fibres may be employed to improve the mechanic characteristics of the composite in coconut shell concrete [5]. However, just like the previous literature Blazy and Blazy [6], the torsional behaviour is not considered.

On the other hand, Usman et al. [57] carried out influential research on the torsional strength of ferrocement by introducing polypropylene (PP) fibres. The primary study was based on experiments conducted on the torsional strength of ferrocement. This investigation introduced the varying proportion of PP fibres to the ferrocement mortar. The amount of PP fibres were added from 0%, 0.3%, 0.6% and 0.9%. For this investigation, a torsion test system was devised to guarantee that torsional force was applied uniformly at both endpoints. Their results showed that PP fibres impacted 67.2 percent of the variance of the torsional strength of ferrocement. The torsional strength enhancement contributed around 39 percent to ferrocement specimens compared to standard ferrocement without added PP fibres. The interaction between the PP fibre and mesh volume was only 2.3% [57].

In addition, Zhou et al. [38] evaluated the effects of fibres on GFRP’s torsional characteristics of reinforced concrete beams. The polypropylene (PP), used to increase the beams’ mechanical qualities, was integrated into the concrete. A total of eight GFRP bars were tested for pure torsional loads when incorporated into reinforced concrete beams with varied fibre content. The test results showed that the PP fibres could successfully restrict the spread of fractures, and the distance and breadth of the cracks decreased. The torsional strength of the reinforced concrete (FRC) beams and of the cementitious composite (ECC) designed were 14% and 50% respectively, which are higher than normal beams within the permissible crack width of 0.7 mm. Furthermore, the larger the fibre content, the higher the torsional strength and stiffness of the beams. The beams were increased by the intensity of the use of the stirrups via the fibres by 1.5 percent [38]. Table 8 provides a comparison of numerous experimental studies on synthetic fibre reinforced concrete.

### 5.3. Hybrid Fibre Reinforced Concrete

Hybrid fibre reinforced concrete, which involves more than one fibre type, was studied by Saravanakumar et al. [58]. The yield strength and torsional behaviour of hybrid fibre reinforced concrete beams were studied through experimental research. Steel fibres and fibre glass were employed to cast the specimens at varied levels of fibre volume, such as 0% 0.5%, 1% and 1.5% concerning the volume of concrete. The test findings showed that with the inclusion of hybrid fibres up to 1 percent in concrete volume, the mechanical performance of the concrete was largely enhanced. In hybrid fibre reinforced concrete, the torsional strength was enhanced in the cracking condition, depending on the amount of the concrete fibre component [59].

The torsional behaviour of the fibre additions was constantly improved with the HFRC beams. In the 1.5 percent HFRC beam, the maximum increase was 36 percent. Failure in the HFRC beam was slowly reached and the specimen sustained the load for a considerable duration after the initial cracking. The results of the torsional behaviour are shown in Table 8.

**Figure 5:**
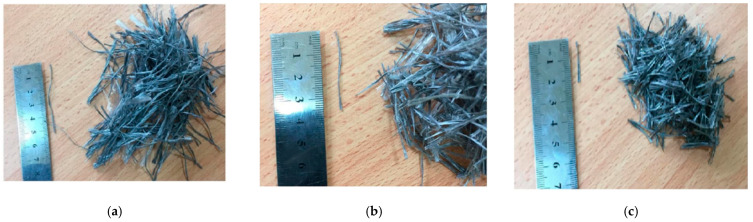
Different lengths of fibres used: (**a**) 57 mm; (**b**) 38 mm; (**c**) 19 mm.

In order to evaluate the torsional resistance of hollow beams’ strength with various kinds of fibre, Hassan et al. [59] took it a step further. In their study, 1 percent of fibre volume fraction and the 13 mm steel fibre ST. F was utilized for three different lengths of synthetic fibre (SY. F), 19, 38 and 57 mm, as shown in Figure 5.

Without the use of fibres, two specimens were produced with the regular concrete mix and four hollow beams were produced with synthetic fibres and steel fibres. The various configurations are shown on Table 9. A novel test procedure was used to put a torsional load on the test materials. At each load interval, the twisting angle of the beams was experimented upon and the first fracture and failure loads were computed. Outcomes showed the overall performance under torsional stress compared to control beam compliance with SY. F and ST. F in the reinforced concrete beam (RCB). This improvement was related to the type and length of the fibre. The test beams reinforced with ST. F and SY were almost identical in length to 19 and 37 mm in the first cracking load. The synthetic fibre with a length of 55 mm showed the maximum initial cracking torque among the other evaluated specimens. With the fibre length increasing from SY. F, for beams with 19 mm, 37 mm and 55 mm of fibre length, the ultimate load capacity rose by 4.7%, 9.4%, and 21.9%, respectively. The ultimate torsional capacity improved by 5.5 percent when using the ST. F reinforcement concrete beam. Therefore, it is advised to use SY.F in typical concrete due to its large influence on the torsional capacity [59]. These findings are further represented on Table 10.

### 5.4. Fibre Reinforced Polymer Concrete

#### 5.4.1. Glass Fibre Reinforced Polymer Concrete

**Figure 6:**
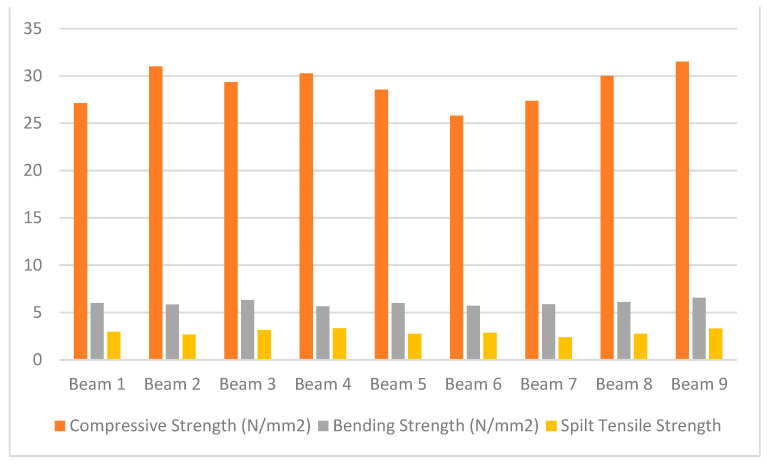
Characteristics of specimens used [60].

Tudu in his research on fibre-glass reinforced concrete, employed nine beams, in which one was labelled the control beam, and the others were reinforced using GFRP. A total of two beams were entirely wrapped in the first series, one with unidirectional GFRP sheets and the other with bidirectional GFRP sheets. The second series comprised of two beams enveloped in 100 mm thick GFRP sheets in the same unidirectional and bidirectional manner as the first series. Two beams in the third series were enveloped with thinner GFRP sheets with a thickness of 50 mm, and two beams in the fourth series were wrapped at a 45-degree angle with GFRP sheets with a thickness of 5 cm. The characteristics of the test sample beams are shown in Figure 6. The torsional moment related to the angle of twist is shown in Table 11.

There was a considerable increase in cracking, ultimate strength, and ultimate twist effect of torque for the strengthened concrete beams enhanced by GFRP sheets. Cracks in the event of enhanced beams have been observed at greater loads. The effectiveness for weight bearing of the enhanced Beam 2 was observed for all beams, which was entirely wrapped with unilateral fibre. The increase in load capacity compared to the control strap was 88.46 percent. Beam 2 and Beam 3 had both collapsed partially without reaching the final load. In the reinforced portion of the sample the failure occurred. The load bearing capacity and torque angle were the finest findings for Beam 8 and Beam 9. In addition, both have the identical GFRP wrapping pattern that is connected with the main beam at an angle of 45 degrees.

Under the experiments with rectangular RC beams reinforced in torsion by GFRPs, several failure mechanisms were found.

Shear failure caused by GFRP rupture is one among them. If the FRP strain achieves its design rupture strain before the concrete strain reaches its maximum effective strain, it is believed that the FRP strips will rupture. Debonding may occur if the substrate is unable to support the FRP force. In this case, strain levels had been limited in order to avoid the GFRP laminate debonding. Dial gauges were used to measure the deflections at L/3, L/2, and 2 L/3 while loads were applied to the beams’ moment arms, which are located at a distance of 0.27 m from the main beam. Section 1 was taken at L/2, while Section 2 was taken at a distance of 300 mm from Sec-1. There was a consistent load arrangement for all of the beams. The load-carrying capability of the control beam and the GFRP reinforced beam were tested. The torsional shear failure of all the beams was discovered.

The un-strengthened cantilever arm conveying the moment on Beam 2 failed, despite the continuous complete wrap of unidirectional fabric around it. Similarly, the cantilever arm conveying the moment of Beam 3, which was continually covered in bi-directional fabric, was found to have failed. It is a partial failure in both circumstances.

Failure occurred in the un-strengthened portion of Beam 4 and Beam 5, which were continually covered with unidirectional and bidirectional 10 cm fabric strips. Shear and torsion combined in the area to cause the breakdown. The fractures in the concrete underneath the textiles began in the area between the strips and spread diagonally. GFRP textiles were not deboned.

The failure patterns of the beams in the third and fourth series were quite similar. The failure was caused by the combination of flexure and torsion causing the GFRP fabric to rip on the bottom face and the 5 cm-wide strips were the starting point for the cracks.

#### 5.4.2. Carbon Fibre Reinforced Polymer Concrete

Tibhe and Rathi [61] presented an experiment that investigated the torsional strengthening of reinforced concrete beams using fibre-reinforced polymers (FRP) material bonded to epoxy. A total of thirty-nine 150 mm × 300 mm × 1200 mm long rectangular beams were produced. Three beams were categorized as control specimens while the remaining beams were divided into two groups with different styles of wrapping. One group had a CFRP fabric wrap and the other had a GFRP fabric wrap. The configurations for CFRP and GFRP were U-enveloped, with vertical bands and edge bands along their full length together with vertical bands. The capacity of the two groups of twisting beams was compared with the torque, twist angle, and factor control and it was noted that the torsional strength of the CFRP twisting beam fabric is more than that of the GFRP torque beam. Table 11, Table 12 and Table 13 expresses findings associated with all carbon fibre and glass fibre beam specimens adopted.

The torsional strength in CFB1 and GFB1 is seen to grow by 60.5% and 47.5%, respectively, with comparison to the control beam. The torsional capacity of CFB6 and GFB6 is the largest and gradually declines for CFB5, CFB4, CFB3, CFB1, and CFB2, and a similar trend was found for CFB beams. The CFB beam has the highest increase in torsional capacity of 101.8 percent, whereas the GFB beam has an increase of 83.49 percent. Furthermore, the minimal increase in torsional capacity of a CFB beam is 40.02 percent and 8.76 percent for a GFB beam. When we compare a CFRP bonded RC beam to a GFRP bonded RC beam, we find that the CFRP fabric has a higher torsional strength than the GFRP fabric. The debonding of CFRP and GFP and the crushing of concrete may have resulted in the GFRP’s and GFP’s failures. We may also remark that the crack width reduces because of the CFRP and GFRP fabric [60].

In this respect, the external enforcement of fibre-reinforced polymer (FRP) may be widely applied to improve the strength requirements in structural systems connected to flexure, shear, and torsion [61].

Similarly, in a study carried out by Chalioris [62], the torsional performance of externally reinforced beams was found to be superior to that of the non-strengthened control specimens. For FRP strips-wrapped beams, failure was a little more delayed than for control specimens, which had already begun to rupture. The unwrapped concrete of the beams, on the other hand, gradually developed torsional diagonal fissures that became wider. A typical experimental curve for an RC beam with FRP strips revealed two separate zones. The varied characteristics of the reaction in these locations indicate the varying nature of the load resisting system in each segment. In general, this suggests that for existing under-reinforced beams exposed to torsion, epoxy-bonded FRP strips may be used to enhance the structure.

#### 5.4.3. Aramid Fibre Reinforced Polymer Concrete

Kandekar et al. [3] presented the torsional behaviour of an aramid fibre reinforced concrete (RC) beam of C30 grade concrete. The aramid fibre was employed to enhance the twisting resistance of the RC beam, as an externally connected reinforcement utilized for improving its flexural strength.

For the torsional strength, the RC beam reinforced with aramid fibre was tested with lever arms that have the same static load and impart the same torque to the beam. The beams had a 150 mm × 300 mm cross-section and measured 1 m long. In total, three torsional strengthening beams and nine normal beams were designed. The beam was developed torsion according to IS456-2000 torsion specifications. The study analysed the characteristics of such typical beams in aramid fibre for torsion. Such reinforced beams’ torsional moments were compared to those manufactured according to new design regulations. The investigation was limited to aramid fibre completely wrapped and wrapped on three faces of the beam with epoxy resin in strips with a width of 100 mm in a U-shape form.

Experimental Ultimate torsional moments & torque at first cracking, twisting angle and form of the twisted beams have shown that fully enveloped reinforced concrete beams provide better torsional capacity than the control specimens and U wrapped beams and that the torsional capacity of the beams enveloped in the strips showed significant improvement. When compared to a controlled specimen, reinforced concrete beams enhanced with completely enveloped aramid fibre took 140 percent higher torque at first fracture and the ultimate torsional moment. When compared to a controlled specimen, reinforced concrete beams enhanced with 100 mm wide aramid fibre strips took 80 percent higher moment at first fracture and ultimate torque. Reinforced concrete beams with 100 mm wide bands using aramid fibre require 80% longer time when compared to the controlled beam at the first and last torsional moment. Beams tend to transfer stress at an increased torsion angle after the first crack. It is therefore a straightforward way to reinforce RC beams. Table 11, Table 12 and Table 13 provides a comparison of different experimental studies on fibre reinforced polymer concrete.

## 6. Conclusions

Fibre reinforced concrete is a combined mixture that contains fibres in an ordered or randomly dispersed manner in the cement matrix. Its qualities are plainly determined by how well stress is redistributed between the matrix and the fibres. From previous studies the following can be deduced:

- Fibre reinforced concrete requires special mixing conditions to mitigate fibre segregation, the balling effect, and the difficulty of consistently amalgamating the components. Increases in aspect ratio, volume fraction, and coarse aggregate quantity and size exacerbate the challenges and balling propensity. Steel fibres with a more than 2% composition in volume and ratio of the length to the diameter greater than 100 are difficult to blend. Fibres used for concrete production must be evenly distributed in the mixture; proper mixing may be accomplished through adding the fibres before the water. When using a laboratory mixer, delivering the fibres through a wire mesh sieve will aid in equal fibre dispersion.
- For effective stress transmission, the matrix of the coefficient of elasticity must be significantly less than that of the fibre. Low modulus fibres, such as nylon 66, polypropylene and acrylic, are less inclined to boost torsional capacity, but they can aid in the intake of huge amounts of energy and result in increased hardness and resilience. Steel, glass, and carbon fibres with a high modulus increase the strength and rigidity of the composite. The efficacy of stress transmission from the matrix to the fibre is also determined by the interfacial connection between the matrix and the fibre. A strong bond is required to increase concrete’s torsional strength.
- The orientation of fibres is also important for improving concrete’s torsional strength. The fibres were found to have high torsional strength and toughness when they were oriented consistently.
- To avoid a significant drop in composite strength, the coarse aggregate size should be limited to 10 mm. Fibres also serve as aggregates. Despite their basic shape, they have a complicated impact on the characteristics of the concrete mix. The fibre distribution and orientation and the features of the composite are controlled by an inter-particle interaction between fibres and aggregates. Friction-reducing admixtures and admixtures that increase the mix’s cohesion can make a big difference.
- The aspect ratio of the fibre is another crucial feature that determines the composite’s characteristics and behaviour. It has been discovered that increasing the aspect ratio up to 75 raises the ultimate strength of concrete linearly. Relative torsional strength and toughness decline in the region of 75.

However, the application of fibres to recycled self-compacting concrete in various forms must be researched to determine its practicality in order to make greener concrete more attainable. Furthermore, the application of natural fibre reinforced polymer in torsion resistance remains unexplored. Hence, further research is encouraged in this area to enhance eco-friendly construction and rehabilitation works.

## Figures and Tables

**Table 1:** Methods of preparation of steel fibre reinforced concrete from previous studies.

References	Specimens	Beam Length	Beam Width	Beam Depth	Fibre Type	Aspect Ratio	Specific Gravity	Supplied Steel Dosage	Area of Steel at Bottom	Area of Steel at Top	Area of Steel for Shear Links	Cu, Concrete (N/mm^2^)	Type of Cement	W/C	Mix Proportion	Size of Coarse Agreggates	Admixtures
Amin and Bentz [37]	T-0-8	1600	200	280					603.000	157.000	251.000	42.300					
	T-0-10								603.000	157.000	393.000	42.300					
	T-30-8				Steel (RC-65/35-BN cold drawn wire fibers 1345 MPa)	54.5	31.200	0.38	603.000	157.000	251.000	42.300					
	T-0-10					54.5	31.200	0.38	603.000	157.000	393.000	42.300					
Facconi et al. [20]	TB1-PC	2700	300	300					509.000	509.000		31.700	CEM I 42.5R	0.5	1:2.84:1.95	4–12 mm	Superplasticizerof 0.37 L/m^3^
	TB2-PC-ST								509.000	509.000	283.000	31.700					Superplasticizerof 0.37 L/m^3^
	TB3-SFRC25				Hooked end Steel	85.7	25.000	0.32	509.000	509.000		31.700					Superplasticizerof 0.74 L/m^3^
	TB4-SFRC25				Hooked end Steel	85.7	25.000	0.32	509.000	509.000		31.700					Superplasticizerof 0.74 L/m^3^
	TB5-SFRC50				Hooked end Steel	85.7	50.000	0.63	509.000	509.000		31.700					Superplasticizerof 1.85 L/m^3^
	TB6-SFRC50				Hooked end Steel	85.7	50.000	0.63	509.000	509.000		31.700					Superplasticizerof 1.85 L/m^3^
Patil et al. [34]	FR0	2000	150	150					101.000	101.000	162.000	20.000	OPC of 53 MPa	0.45	1:1.5:3	4–20 mm	
	FR0.5				Hooked end Steel	30–250	12.500	0.50	101.000	101.000	162.000	20.000					
	FR1				Hooked end Steel	30–250	25.000	1.00	101.000	101.000	162.000	20.000					
	FR1.5				Hooked end Steel	30–250	37.500	1.50	101.000	101.000	162.000	20.000					
lau et al. [39]	F1	1300	150	250	The Double end hooked Dramix 5D 65/60BG steel fibers	66.7	12.500	0.50				56.000	Fly ash, silica, fumes and ground granulated furnace slag	0.45	1:1.23:1.93	4–7 mm	Alkali activators
	F2					66.7	12.500	0.50				56.000					
	F3					66.7	12.500	0.50				56.000					
	R1						12.500	0.50	157.000	157.000	393.000	56.000					
	R2						12.500	0.50	157.000	157.000	393.000	56.000					
	R3						12.500	0.50	157.000	157.000	393.000	56.000					
	RF2					66.7	12.500	0.50	157.000	157.000	393.000	56.000					
	RF3					66.7	12.500	0.50	157.000	157.000	393.000	56.000					
George & Sofi [56]	NWC-0%	1100	100	150					101.000	101.000	207.000	32.200	OPC of 53 grade	0.5	1:77:3.1	12.5–20 mm	
	NWC-0.5%				Grooved steel fibres	50		0.50	101.000	101.000	207.000	44.200					
	NWC-0.75%					50		0.75	101.000	101.000	207.000	58.970					
	NWC-1%					50		1.00	101.000	101.000	207.000	59.970					
	CSC-0%							-	101.000	101.000	207.000	30.167	OPC of 53 grade and Silica fume	0.31	1:1.37:0.91	12.5 mm	Superplasticizer (Cer Hyperplast XR-W40) at 0.7% by wt. of binder.
	CSC-0.5%				Grooved steel fibres	50		0.50	101.000	101.000	207.000	35.360					
	CSC-0.75%					50		0.75	101.000	101.000	207.000	35.600					
	CSC-1%					50		1.00	101.000	101.000	207.000	36.600					

**Table 2:** Methods of preparation of synthetic fibre reinforced concrete from previous studies.

References	Specimens	Beam Length	Beam Width	Beam Depth	Fibre Type	Aspect Ratio	Specific Gravity	Supplied Steel Dosage	Volume of Reinforcement to Volume of Composite	Cu, Concrete (N/mm^2^)	Type of Cement	W/C	Mix Proportion	Size of Coarse Agreggates	Admixtures
Usman et al. [57]	S1-0%	250	25	80	Polypropylene				0.013		Ferrocement				
	S2-0%								0.025						
	S3-0%								0.038						
	S4-0.3%							0.30	0.013						
	S5-0.3%							0.30	0.025						
	S6-0.6%							0.30	0.038						
	S7 -0.6%							0.60	0.013						
	S8-0.6%							0.60	0.025						
	S9-0.9%							0.60	0.038						
	S10 -0.9%							0.90	0.013						
	S11-0.9%							0.90	0.025						
	S12-0.9%							0.90	0.025						
Zhou et al. [38]	S-1	1800	150	200	OC				0.021	42.500	OPC	0.4	1:1.46:0.86	4–15 mm	
	S-2								0.041	42.500			1:1.46:0.86		
	F-1				FRC with Polypropylene	1000	9.1	1.00	0.021	40.200	OPC and Fly ash at 214 kg/m^3^	0.57	1:1.18:0.79		Plasticizer at 4.25 kg/m^3^
	F-2					1000	9.1	1.00	0.041	40.200			1:1.18:0.80		
	F-3					1000	9.1	1.00	0.021	40.200			1:1.18:0.81		
	F-4					1000	9.1	1.00	0.041	40.200			1:1.18:0.82		
	E-1					1000	13.65	1.50	0.021	32.800	OPC and Fly ash at 265 kg/m^3^	0.54	1:1		
	E-2					1000	13.65	1.50	0.040	32.800			1:1		

**Table 3:** Methods of preparation of hybrid fibre reinforced concrete from previous studies.

References	Specimens	Beam Length	Beam Width	Beam Depth	Fibre Type	Aspect Ratio	Specific Gravity	Supplied Steel Dosage	Area of Steel at Bottom	Area of Steel at Top	Area of Steel for Shear Links	Cu, Concrete (N/mm^2^)	Type of Cement	W/C	Mix Proportion	Admixtures
Saravanakumar et al. [58]	HFRC0.0	1200	150	230					151.000	101.000	335.000	21.500	OPC 53 grade		1:1.41:3.09	Sulphonated napthalene polymer based super plasticizer SP 430
	HFRC0.5				Corrugated circular Steel fibres of size 1 mm × 36 mm with avg. pitch of 8 mm and rise of 2 mm; Avg. ultimate strenght of 600 Mpa and Modulus of elasticity of 210,000 MPa and Glass fibres having diameter of 0.0153 mm, relative density of 2.7 g/m^3^, ultimate tensile strength of 2900 MPa, elastic modulus of 73.4 MPa.				151.000	101.000	335.000	23.300				
	HFRC1.0								151.000	101.000	335.000	26.400				
	HFRC1.5								151.000	101.000	335.000	24.300				
Hassan et al. [59]	C (Solid beam without fiber)	1000	150	150											1:1.56:2.44	
	H (Hollow beam (75 mm dia hollow) without fibre)				Synthetic fibers of lengths 19 mm, 38 mm and 57 mm, and steel fibers of length 13 mm and diameter ranging from 0.2 to 0.3 mm				157.000	157.000	400.000		OPC	0.32		Superplasticizer of 1/100 kg cement
	HS (Hollow beam reinf. With ST. F								157.000	157.000	400.000					
	H20 (Hollow beam reinf. With SY. F of 19 mm length)								157.000	157.000	400.000					
	H30 (Hollow beam reinf. With SY.F 37 mm length)								157.000	157.000	400.000					
	H50 (Hollow beam with SY.F of 55 mm of length)								157.000	157.000	400.000					

**Table 4:** Methods of preparation of glass fibre reinforced polymer concrete from previous studies.

References	Specimens	Beam Length	Beam Width	Beam Depth	Fibre Type	Area of Steel at Bottom	Area of Steel at Top	Area of Steel for Shear Links	Cu, Concrete (N/mm^2^)	Type of Cement	W/C	Mix Proportion
Tudu [60]	Beam No. 1 (Control Beam)	1650	150	250		402.000	157.000	188.000	27.110	OPC	0.5	1:1.8:3.6
	Beam No. 2 (Uni-GFRP continuous fully wrap)				Glass fiber reinforced polymer (GFRP)	402.000	157.000	188.000	31.000			1:1.8:3.6
	Beam No. 3 (Bi-GFRP Continuous fully wrap)					402.000	157.000	188.000	29.340			1:1.8:3.6
	Beam No. 4 (10 cm Uni-GFRP strips wrap)					402.000	157.000	188.000	30.250			1:1.8:3.6
	Beam No. 5 (10 cm Bi-GFRP)					402.000	157.000	188.000	28.530			1:1.8:3.6
	Beam No. 6 (5 cm Uni-GFRP strips wrap)					402.000	157.000	188.000	25.780			1:1.8:3.6
	Beam No. 7 (5 cm Bi-GFRP strips wrap)					402.000	157.000	188.000	27.360			1:1.8:3.6
	Beam No. 8 (5 cm Uni-GFRP strips wrap at 45 degrees)					402.000	157.000	188.000	30.000			1:1.8:3.6
	Beam No. 9 (5 cm Bi-GFRP strips wrap at 45 degrees)					402.000	157.000	188.000	31.500			1:1.8:3.6

**Table 5:** Methods of preparation of carbon fibre reinforced concrete from previous studies.

References	Specimens	Beam Length	Beam Width	Beam Depth	Fibre Type	Aspect Ratio	Specific Gravity	Supplied Steel Dosage	Area of Steel at Bottom	Area of Steel at Top	Area of Steel for Shear Links	Cu, Concrete (N/mm^2^)	Type of Cement	W/C	Mix Proportion	Size of Coarse Agreggates
Tibhe & Rathi [61]	Control Beam	1200	150	300					339.000	101.000	203.000	30.000	OPC 53 grade Ultra tech	0.45	1:1.76:2.77	4–20 mm
	CFB1				Carbon fibre reinforced polymer (CFRP) with young’s modulus of 70–90, tensile strength of 2400–5100 MPa, strain at failure of 0.5–1.73 and density of 1.85–1.9 andGlass fibre reinforced polymer (GFRP) with young modulus of 390–760 GPa, tensile strength of 3000–4800 MPa, strain at failure of 3.5–5.5% and density of 2.5–2.6 g/cm^2^)				339.000	101.000	203.000	30.000				
	GFB1								339.000	101.000	203.000	30.000				
	CFB2								339.000	101.000	203.000	30.000				
	GFB2								339.000	101.000	203.000	30.000				
	CFB3								339.000	101.000	203.000	30.000				
	GFB3								339.000	101.000	203.000	30.000				
	CFB4								339.000	101.000	203.000	30.000				
	GFB4								339.000	101.000	203.000	30.000				
	CFB5								339.000	101.000	203.000	30.000				
	GFB5								339.000	101.000	203.000	30.000				
	CFB6								339.000	101.000	203.000	30.000				
	GFB6								339.000	101.000	203.000	30.000				
Chalioris [62]	Ra-c				Carbon fibre reinforced polymer (CFRP) with thickness 0.11 mm, elastic modulus of 230 GPa, Ultimate tensile strength of 3900 MPa, elongation at failure 1.5%mm/m							27.500				
	Ra-Fs150(2)	1600	150	300					101.000	101.000		27.500				
	Ra-S								101.000	101.000	226.000	27.500				
	Ra-SFs150(2)	1600	150	300					101.000	101.000	226.000	27.500				
	Rb-c								101.000	101.000		28.800				
	Rb-Fs200(1)	1600	200	300					101.000	101.000		28.800				
	Rb-S								101.000	101.000	283.000	28.800				
	Rb-SFs200(1)	1600	200	300					101.000	101.000	283.000	28.800				

**Table 6:** Methods of preparation of aramid fibre reinforced concrete from previous studies.

References	Specimens	Beam Length	Beam Width	Beam Depth	Fibre Type	Aspect Ratio	Specific Gravity	Supplied Steel Dosage	Area of Steel at Bottom	Area of Steel at Top	Area of Steel for Shear Links	Cu, Concrete (N/mm^2^)	W/C	Mix Proportion
Kandekar et al. [3]	C1 (Controlled beam)	1000	150	300	Aramid				151.000	101.000	335.000	30.000	0.45	1:2.14:3.54
	C2 (Conctrolled Beam)								151.000	101.000	335.000	30.000		
	C3 (Controlled Beam)								151.000	101.000	335.000	30.000		
	T1 (Designed ror Torsion)								151.000	101.000	335.000	30.000		
	T2 (Designed ror Torsion)								151.000	101.000	335.000	30.000		
	T3 (Designed ror Torsion)								151.000	101.000	335.000	30.000		
	F1 (fully wrapped beam)					Aramid fiber properties; weave style is plain, Areal weight of fabric is 300 g/m^2^, standard with is 1000 mm, dry fabric thickness is 0.25 mm; Tensile strength is 2400–3600 MPa, Tensile Modulus is 60–120 GPa, Elongation percentage 2.2–4.4%			151.000	101.000	335.000	30.000		
	F2 (fully wrapped beam)								151.000	101.000	335.000	30.000		
	F3 (fully wrapped beam)								151.000	101.000	335.000	30.000		
	S1 (Wrapped with strip)								151.000	101.000	335.000	30.000		
	S2 (wrapped with strip)								151.000	101.000	335.000	30.000		
	S3 (wrapped with strip)								151.000	101.000	335.000	30.000		

**Table 7:** Comparison of different experimental studies for steel fibre reinforced concrete.

References	Specimens	Beam Width	Beam Depth	Longitudinal Rebar Ratio (%)	Fibre Volume Fraction (%)	Fibre Volume Fraction/0.25%	Ultimate Torque	Ultimate Torque/Torque at First Crack	Ultimate Twist/Twist at First Crack	Torsional Strength Increase (Based on Optimal and Relevant Strengthening Configuration)%
Amin and Bentz, 2018	T-0-8	200.00	280.00	1.36			16.00			37.00
	T-0-10			1.36			16.00			
	T-30-8			1.36	0.38	1.52	21.00			
	T-30-10			1.36	0.38	1.52	23.00			
Facconi et al. 2021	TB1-PC	300.00	300.00	1.13			16.18	1.30	1.60	55.43
	TB2-PC-ST			1.13			20.84	1.59	22.97	
	TB3-SFRC25			1.13	0.32	1.28	27.32	2.04	17.48	
	TB4-SFRC25			1.13	0.32	1.28	22.94	1.91	9.40	
	TB5-SFRC50			1.13	0.63	2.52	26.94	1.94	15.19	
	TB6-SFRC50			1.13	0.63	2.52	24.63	1.79	8.99	
Patil et al. 2016	FR0	150.00	150.00	0.90			2.26			72.00
	FR0.5			0.90	0.50	2.00	2.50			
	FR1			0.90	1.00	4.00	2.96			
	FR1.5			0.90	1.50	6.00	3.07			
lau et al. [39]	F1	150.00	250.00		0.50	2.00	8.80	1.31	3.29	25.00
	F2				0.50	2.00	7.70	1.00	1.00	
	F3				0.50	2.00	8.30	1.04	1.07	
	R1			0.84	0.50	2.00	9.80	1.72	3.12	
	R2			0.84	0.50	2.00	7.60	1.31	1.30	
	R3			0.84	0.50	2.00	7.70	1.03	1.13	
	RF2			0.84	0.50	2.00	10.10	1.40	3.33	
	RF3			0.84	0.50	2.00	10.80	1.38	3.59	
George & Sofi [56]	NWC-0%	100.00	150.00	1.35			2.55	1.19		155.37
	NWC-0.5%			1.35	0.50	2.00	4.05	1.06		
	NWC-0.75%			1.35	0.75	3.00	6.51	1.09		
	NWC-1%			1.35	1.00	4.00	6.22	1.11		
	CSC-0%			1.35			4.80	1.10		
	CSC-0.5%			1.35	0.50	2.00	5.33	1.04		
	CSC-0.75%			1.35	0.75	3.00	7.25	1.08		
	CSC-1%			1.35	1.00	4.00	6.51	1.06		

**Table 8:** Comparison of different experimental studies for synthetic fibre reinforced concrete.

References	Specimens	Beam Width	Beam Depth	Longitudinal Rebar Ratio (%)	Fibre Volume Fraction (%)	Fibre Volume Fraction/0.25%	Ultimate Torque	Ultimate Torque/Torque at First Crack	Torsional Strength Increase (Based on Optimal and Relevant Strengthening Configuration) %
Usman et al. [57]	S1-0%	25.00	80.00	1.26			0.05		39.00
	S2-0%			2.51			0.05		
	S3-0%			3.77			0.05		
	S4-0.3%			1.26	0.30	1.20	0.05		
	S5-0.3%			2.51	0.30	1.20	0.06		
	S6-0.3%			3.77	0.30	1.20	0.07		
	S7-0.6%			1.26	0.60	2.40	0.06		
	S8-0.6%			2.51	0.60	2.40	0.07		
	S9-0.6%			3.77	0.60	2.40	0.08		
	S10-0.9%			1.26	0.90	3.60	0.05		
	S11-0.9%			2.51	0.90	3.60	0.05		
	S12-0.9%			2.51	0.90	3.60	0.06		
Zhou et al. [38]	S-1	150.00	200.00	2.05			5.45	1.89	
	S-2			4.10			5.83	2.10	
	F-1			2.05	1.00	4.00	6.67	2.32	15.18
	F-2			4.10	1.00	4.00	7.00	2.32	
	F-3			2.05	1.00	4.00	5.70	2.05	
	F-4			4.10	1.00	4.00	6.43	2.52	
	E-1			2.05	1.50	6.00	8.23	3.61	49.23
	E-2			4.00	1.50	6.00	8.70	3.95	

**Table 9:** Sample IDs [59].

Specimen	Configuration
C	Solid beam specimen without fibre
H	Hollow beam specimen without fibre
HS	Hollow beam specimen reinforced with ST. F
H20	Hollow beam specimen reinforced with SY. F of 19 mm Length
H30	Hollow beam specimen reinforced with SY. F 37 mm Length
H50	Hollow beam specimen reinforced with SY. F of 55 mm Length

**Table 10:** Comparison of different experimental studies for hybrid fibre reinforced concrete.

References	Specimens	Beam Width	Beam Depth	Longitudinal Rebar Ratio (%)	Fibre Volume Fraction (%)	Fibre Volume Fraction/0.25%	Ultimate Torque	Ultimate Torque/Torque at First Crack	Ultimate Twist/Twist at First Crack	Torsional Strength Increase (Based on Optimal and Relevant Strengthening Configuration) %
Saravanakumar et al. [58]	HFRC0.0	150.00	230.00	0.73	-	-	4.70	1.15		36.17
	HFRC0.5			0.73	0.50	2.00	5.20	1.16		
	HFRC1.0			0.73	1.00	4.00	6.10	1.17		
	HFRC1.5			0.73	1.50	6.00	6.40	1.17		
Hassan et al. [59]	C (Solid beam without fibre)	150.00	150.00				6.40	1.60	2.52	21.88
	H (Hollow beam (75 mm dia hollow) without fibre)			1.40			6.20	1.77	3.26	
	HS (Hollow beam reinf. with 13 mm ST. F			1.40	0.25	1.00	6.75	1.59	3.76	
	H20 (Hollow beam reinf. with SY. F of 19 mm length)			1.40	0.50	2.00	6.96	1.64	3.27	
	H30 (Hollow beam reinf. with SY.F 37 mm length)			1.40	0.75	3.00	7.00	1.65	3.27	
	H50 (Hollow beam with SY.F of 55 mm of length)			1.40	1.00	4.00	7.80	1.73	4.91	

**Table 11:** Experimental findings on glass fibre reinforced polymer concrete.

References	Specimens	Beam Width	Beam Depth	Longitudinal Rebar Ratio (%)	Fibre Strip Width (mm)	Fibre Thickness (mm)	Ultimate Torque	Ultimate Torque/Torque at First Crack	Ultimate Twist/Twist at First Crack	Torsional Strength Increase (Based on Optimal and Relevant Strengthening Configuration) %
A. Glass Fibre Reinforced Polymer										
Tudu [60]	Beam No. 1 (Control Beam)	150.00	250.00	1.49	-		35.10	1.63	2.00	54.62
	Beam No. 2 (Uni-GFRP continuous fully wrap)			1.49	25.00	1.50	66.13	2.72	1.34	
	Beam No. 3 (Bi-GFRP Continuous fully wrap)			1.49	25.00	3.00	56.70	1.62	1.28	
	Beam No. 4 (10 cm Uni-GFRP strips wrap)			1.49	25.00	100.00	48.60	1.80	2.25	
	Beam No. 5 (10 cm Bi-GFRP)			1.49	25.00	100.00	58.05	1.79	3.16	
	Beam No. 6 (5 cm Uni-GFRP strips 90o wrap)			1.49	25.00	50.00	46.98	2.18	3.78	
	Beam No. 7 (5 cm Bi-GFRP strips 90o wrap)			1.49	25.00	50.00	58.32	1.96	2.25	
	Beam No. 8 (5 cm Uni-GFRP strips wrap at 45 degrees)			1.49	25.00	50.00	54.00	2.22	2.05	
	Beam No. 9 (5 cm Bi-GFRP strips wrap at 45 degrees)			1.49	25.00	50.00	54.54	1.84	2.51	

**Table 12:** Experimental findings on carbon fibre reinforced polymer concrete.

References	Specimens	Beam Width	Beam Depth	Longitudinal Rebar Ratio (%)	Fibre Strip Width (mm)	Fibre Thickness (mm)	Ultimate Torque	Ultimate Torque/Torque at First Crack	Ultimate Twist/Twist at First Crack	Torsional Strength Increase (Based on Optimal and Relevant Strengthening Configuration) %
B. Carbon Fibre Reinforced Polymer										
Tibhe & Rathi [61]	ControlBeam	150.00	300.00	0.98			12.22	2.33	18.12	101.80
	CFB1			0.98	150.00		19.61	2.35	16.33	
	GFB1			0.98	150.00		18.02	2.40	15.19	
	CFB2			0.98	150.00		17.11	2.12	15.41	
	GFB2			0.98	150.00		15.40	2.21	15.63	
	CFB3			0.98	150.00		17.12	2.04	15.81	
	GFB3			0.98	150.00		13.29	1.84	15.08	
	CFB4			0.98	150.00		21.87	2.34	15.08	
	GFB4			0.98	150.00		19.02	2.48	15.29	
	CFB5			0.98	150.00		22.47	2.24	12.93	
	GFB5			0.98	150.00		20.62	2.41	15.01	
	CFB6			0.98	150.00		24.66	2.02	11.75	
	GFB6			0.98	150.00		22.42	2.13	13.15	
Chalioris [62]	Ra-c (no stirrups)						2.39	1.00		79.67
	Ra-Fs150(2) (no stirrups)	150.00	300.00	0.45	150.00	0.22	3.02	1.36		
	Ra-S (with stirrups)						2.41	1.07		
	Ra-SFs150(2) (with stirrups)	150.00	300.00	0.45	150.00	0.22	4.33	1.84		
	Rb-c (no stirrups)						6.95	1.00		
	Rb-Fs200(1) (no stirrups)	200.00	300.00	0.34	200.00	0.11	9.32	1.38		
	Rb-S (with stirrups)						7.15	1.04		
	Rb-SFs200(1) (with stirrups)	200.00	300.00	0.34	200.00	0.11	9.80	1.41		

**Table 13:** Experimental findings on aramid fibre reinforced polymer concrete.

References	Specimens	Beam Width	Beam Depth	Longitudinal Rebar Ratio (%)	Fibre Strip Width (mm)	Fibre Thickness (mm)	Ultimate Torque	Ultimate Torque/Torque at First Crack	Torsional Strength Increase (Based on Optimal and Relevant Strengthening Configuration) %
Kandekar & Talikoti [3]	C1 (Controlled beam)	150.00	300.00	0.56			3.30	1.22	
	C2 (Conctrolled Beam)			0.56			2.90	1.21	
	C3 (Controlled Beam)			0.56			3.00	1.20	
	T1 (Designed for Torsion)			0.78			7.80	1.22	166.67
	T2 (Designed for Torsion)			0.78			8.50	1.23	
	T3 (Designed for Torsion)			0.78			8.50	1.25	
	F1 (fully wrapped beam)			0.56	1000.00	0.25	8.95	1.42	173.66
	F2 (fully wrapped beam)			0.56	1000.00	0.25	8.50	1.20	
	F3 (fully wrapped beam)			0.56	1000.00	0.25	8.00	1.16	
	S1 (Wrapped with strip)			0.56	1000.00	0.25	6.65	1.28	102.69
	S2 (wrapped with strip)			0.56	1000.00	0.25	6.00	1.25	
	S3 (wrapped with strip)			0.56	1000.00	0.25	6.20	1.24	
